# Overexpression of X Intrinsic Protein 1;1 in *Nicotiana tabacum* and Arabidopsis reduces boron allocation to shoot sink tissues

**DOI:** 10.1002/pld3.143

**Published:** 2019-06-05

**Authors:** Manuela Desiree Bienert, Beatriz Muries, Delphine Crappe, François Chaumont, Gerd Patrick Bienert

**Affiliations:** ^1^ Department of Physiology and Cell Biology Leibniz Institute of Plant Genetics and Crop Plant Research Gatersleben Germany; ^2^ Louvain Institute of Biomolecular Science and Technology UCLouvain Louvain‐la‐Neuve Belgium

**Keywords:** aquaporin, boron, metalloid transport, plant nutrition, transporter, X Intrinsic Protein

## Abstract

Major Intrinsic Proteins (MIP) are a family of channels facilitating the diffusion of water and/or small solutes across cellular membranes. X Intrinsic Proteins (XIP) form the least characterized MIP subfamily in vascular plants. XIPs are mostly impermeable to water but facilitate the diffusion of hydrogen peroxide, urea and boric acid when expressed in heterologous expression systems. However, their transport capabilities in planta and their impact on plant physiology are still unknown. Here, we demonstrated that overexpression of NtXIP1;1 in *Nicotiana tabacum* by the En2pPMA4 or the 35S CaMV promoter and in Arabidopsis, which does not contain any *XIP* gene, by the 35S CaMV promoter, resulted in boron (B)‐deficiency symptoms such as death of the shoot apical meristem, infertile flowers, and puckered leaves. Leaf B concentrations in symptomatic tissues and B xylem sap concentrations were lower in the overexpressors than in control plants. Importantly, expression of *NtXIP1;1* under the control of the *AtNIP5;1* promoter complemented the B deficiency phenotype of the *Atnip5;1* knockout mutant, defining its ability to act as a boric acid channel *in planta*. Protein quantification analysis revealed that NtXIP1;1 was predominantly expressed in young B‐demanding tissues and induced under B‐deficient conditions. Our results strongly suggest that NtXIP1;1 plays a role in B homeostasis and its tissue‐specific expression critically contributes to the distribution of B within tobacco.

## 1 INTRODUCTION

Major Intrinsic Proteins (MIPs) are membrane channels found in all kingdoms of life. They assemble as tetramers in various cellular membranes whereas each monomer represents an individual functional unit. Initially, MIPs have been identified as physiologically important water channels, the so‐called aquaporins (Preston, Carroll, Guggino, & Agre, 1992). However, research in the past decades pointed out that individual isoforms are capable of facilitating the transport of further small solutes ranging from the signaling molecule hydrogen peroxide (Bienert et al., 2007) to the essential plant nutrients boron (B) and silicon (Ma et al., 2006; Takano et al., 2006). Moreover, it has been demonstrated that MIPs play essential roles in the uptake, translocation and cell‐to‐cell transport of these compounds. Selectivity is believed to be mainly determined by physico‐chemical channel pore characteristics at the aromatic arginine (ar/R) region, constituted of four amino acid residues, one of transmembrane helix 2, one of transmembrane helix 5 and two from loop E. Additionally, two NPA motifs, consisting of the highly conserved asparagine‐proline‐alanine signature, form a restrictive site which is important for hydrogen ion exclusion through the channel (Murata et al., 2000). Compared to 13 isoforms in humans (Agre & Kozono, 2003), a relatively large number of isoforms are expressed in plants e.g. 35 MIPs in *Arabidopsis thaliana* (Johanson et al., 2001), 71 in cotton (Park, Scheffler, Bauer, & Campbell, 2010) and even 121 in *Brassica napus* (Yuan et al., 2017). In vascular plants, MIPs phylogenetically cluster into four to five distinct subfamilies namely the Plasma membrane Intrinsic Proteins (PIPs), Tonoplast Intrinsic Proteins (TIPs), Small basic Intrinsic Proteins (SIPs), Nodulin26‐like Intrinsic Proteins (NIPs) and the X‐Intrinsic Proteins (XIPs). While PIP, TIP, SIP and NIP subfamily members occur in all so far published genomes of land plant taxa, XIPs have most likely been evolutionarily lost in monocots and some eudicots such as *Brassicacea* species (Danielson & Johanson, 2008). Initially, XIPs were discovered in the moss *Physcomitrella patens* (Danielson & Johanson, 2008). Thereafter, XIPs were identified in a number of plant species such as poplar (Gupta & Sankararamakrishnan, 2009), tomato (Sade et al., 2009) or *Nicotiana tabacum* (Bienert, Bienert, Jahn, Boutry, & Chaumont, 2011).

The first molecular characterization of plant XIPs demonstrated that NtXIP1;1 from tobacco is localized in the plasma membrane and that its promoter is widely active throughout the plant, both in roots and shoots (Bienert et al., 2011). In *N. tabacum* and *Nicotiana benthamiana*, the transcript of *XIP1;1* is spliced in two distinct variants, *XIP1;1*α and *XIP1;1*β differing in only one amino acid in length (Ampah‐Korsah et al., 2016, 2017; Bienert et al., 2011). Toxicity growth assays in the heterologous expression systems *Saccharomyces cerevisiae* and *Pichia pastoris*, respectively, suggest that NtXIP1;1α, NtXIP1;1β and NbXIP1;1α function as boric acid channels (Ampah‐Korsah et al., 2016; Bienert et al., 2011). Additionally, NtXIP1;1 was shown to facilitate the membrane diffusion of glycerol, urea and probably hydrogen peroxide upon heterologous expression in *S. cerevisiae* cells and *Xenopus laevis* oocytes. In contrast, the XIPs characterized so far are not able to relevantly channel water (Bienert et al., 2011; Lopez et al., 2016; Noronha et al., 2016).

Phylogenetic comparisons of XIP isoforms of different land plant species identified two major clusters composed of four (Lopez et al., 2012) or five (Venkatesh, Yu, Gaston, & Park, 2015) distinct XIP groups. This diversity in XIP sequences may hint to heterogeneous functions of various isoforms. In addition, to their existence in plants, XIPs have also been identified in protozoan and fungal genomes (Danielson & Johanson, 2008; Gupta & Sankararamakrishnan, 2009). Till date, the physiological role of XIPs remains speculative and data on the endogenous function of any fungal, protozoan or plant isoform are missing.

Analysis of three XIP sequences from *Hevea brasiliensis* revealed that they cluster into two groups (Lopez et al., 2016). Thereof, *HbXIP2;1* is expressed in all vegetative tissues. This rather low, but general expression in the plant body seems common to many analyzed XIPs (Bienert et al., 2011; Kong, Bendahmane, & Fu, 2018). Interestingly, *HbXIP2;1* expression is upregulated specifically during latex tapping and by ethylene treatments. As HbXIP2;1 is able to facilitate the diffusion of glycerol it was speculated that it is potentially involved in maintaining the glycerol to water balance in tapping challenged laticifers (Lopez et al., 2016). However, experimental evidence for that hypothesis remains to be provided. In Sweet Orange and grapevine, transcript abundances of XIPs were altered by drought stress and their potential role in maintaining the osmotic balance is discussed. However, transcript abundance upon drought stress is not relatable from one species to another. Whereas in Sweet Orange *CsXIPs* are upregulated in leaves and downregulated in roots upon drought stress, *VvXIP1* in grapevine is strongly downregulated in leaves of water‐stressed plants (de Paula Santos Martins et al., 2015; Noronha et al., 2016). In *Lotus japonicus*,* LjXIP1* is induced by colonization of the root with arbuscular mycorrhizal fungi but not by rhizobial bacteria, hinting towards a function in the symbiotic interaction (Giovannetti et al., 2012).

Summarizing the current knowledge on plant XIPs, it becomes obvious that molecular characterization efforts for various plant XIPs are confined to developmental‐ and organ‐specific expression analyses and transport selectivity assays in heterologous expression systems. Both approaches resulted in a diffuse diversity of identified substrates and expression patterns which pointed neither to a clear functional nor physiological understanding of this uncharacterized protein channel family. A physiological function of a single XIP isoform *in planta* is pending to be shown. Until now, classical reverse genetic approaches targeting specific XIP isoforms are lacking.

Here, we studied the effects of genetically modulated NtXIP1;1 expression in *N. tabacum* on plant performance. Interestingly, overexpression of NtXIP1;1 in its endogenous host led to abnormal plant and tissue growth. We demonstrated that the overexpression of NtXIP1;1 in *N. tabacum* leads to severe B deficiency symptoms which were rescued by the application of a surplus addition of B. Although protein expression is induced under B deficiency in *N. tabacum* leaves, silencing of *NtXIP1;1* did not result in any apparent phenotype neither under standard nor B‐deficient growth conditions. Overexpressing of NtXIP1;1 in *A. thaliana* also led to B deficiency symptoms comparable to the overexpression phenotype in *N. tabacum*. Interestingly, expression of *NtXIP1;1* under the control of the *AtNIP5;1* promoter rescued the B deficiency phenotype of the *Atnip5;1* knockout mutant, demonstrating that *NtXIP1;1* functions as a boric acid channel in plants. Using the *X. laevis* oocyte system, we quantitatively provided evidence that XIPs do indeed transport boric acid in a direct B uptake assay.

## 2 MATERIALS AND METHODS

### 2.1 Oocyte swelling assay

Oocytes were isolated from adult female *X. laevis* frogs, and placed in a Barth's buffer solution. Oocytes were treated with 0.1% collagenase type B (Roche Diagnostics) in a Ca‐free Barth buffer for 1.5 hr to remove follicular cell layers, washed five times with Barth buffer free of collagenase, and selected according to the size and developmental stage. Selected oocytes were incubated for 1 day in Barth buffer at 18°C. *NtXIP1;1* cRNA with cap analog was synthesized with mMESSAGE mMACHINE High Yield Capped RNA transcription kit (Ambion) according to the manufacturer's instructions using the NtXIP1;1 oocyte pNB1u expression vectors (Bienert et al., 2011). Fifty nanoliters of cRNA (12.5 ng/50 nl) were injected into the selected oocytes. As a negative control, 50 nl of RNase‐free water was injected. After incubation in Barth's buffer at 18°C for 72 hr, the oocytes (*n* = 8–10 oocytes per pool/replicate; 9–15 pools/replicates) were exposed to Barth buffer containing 5 mM ^10^B(OH)_3_ (Sigma Aldrich). After 0, 5, and 20 min exposure, the oocytes were washed three times with Barth buffer containing 5 mM ^11^B(OH)_3_. Samples were dried (65°C) and digested with concentrated nitric acid and the ^10^B/^11^B content was subsequently determined using a sector field high resolution mass spectrometer (HR‐ICP‐MS). Uptake assays were performed with oocytes isolated from three different frogs resulting in consistent results.

### 2.2 Plant material

For sterile culture *N. tabacum* seeds (*cv. Petit Havana SR1*) (Maliga, Sz‐Breznovits, & Márton, 1973) were sterilized using chlorine gas for 30 min and sown onto solid Murashige and Skoog (MS) medium with or without antibiotics (4.4 g/L MS salts with minimal organics [Sigma], 3% sucrose, 0.7% agar, pH 5.8 [KOH]). Seeds were placed in a phytotron (8 hr‐dark‐18°C/16 hr‐light‐24°C). Plantlets were transferred to soil after 10 days. For growth on soil, homozygous seeds were germinated on soil in a phytotron (8 hr‐dark‐18°C/16 hr‐light‐24°C). Plantlets were transferred into individual pots after 10 days of growth.

*Arabidopsis thaliana* wild type (Col‐0) seeds or a T‐DNA insertion line for *AtNIP5;1* (SALK_122287) were surface sterilized with 70% ethanol plus 0.05% Triton X‐100 followed by three washes with 99% ethanol. Dry seeds were sown on half‐strength MS medium (2.2 g/L MS salts with minimal organics [Sigma], 1% sucrose, 0.7% agar, pH 5.8 [KOH]) with or without antibiotic. Seeds were vernalized for 2 days at 4°C. In vitro cultures were grown at 120 μM m^−2^ s^−1^ in a 10 hr light/14 hr dark cycle at 22°C/19°C, respectively. After germination and seedling development, plants were transferred to either soil or sterile culture medium.

### 2.3 Cloning of constructs

For overexpression of NtXIP1;1 in *N. tabacum*, the *NtXIP1;1* coding sequence was cloned into pAUX3131 between the constitutive *NpPMA4* promoter fused to two copies of the 35S enhancer (*En2pPMA4*) and the *tNOS* terminator (Table S1) (De Muynck, Navarre, Nizet, Stadlmann, & Boutry, 2009). The expression cassette was excised from pAUX3131 using I‐SceI and inserted into pPZP‐RCS2‐nptII. For overexpression of *NtXIP1;1* in *A. thaliana* or *N. tabacum*, the *NtXIP1;1* coding sequence was USER‐cloned into pCAMBIA 2300 35Su between the *CaMV 35S* promoter and the *tNOS* terminator (Table S1) (Nour‐Eldin, Hansen, Norholm, Jensen, & Halkier, 2006). For the *Atnip5;1* knockout mutant transformation, a 2,429 bp fragment of the *AtNIP5;1* promoter sequence was amplified (Table S1) and cloned into the pENTR vector of the GATEWAY cloning system (Invitrogen). *NtXIP1;1* was amplified (Table S1) and fused behind the *AtNIP5;1* promoter sequence. The final construct was cloned via the LR reaction into pGWB‐01.

### 2.4 Silencing of NtXIP1;1

Silencing of *NtXIP1;1* in *N. tabacum* was performed applying the artificial microRNA technique (Schwab, Ossowski, Riester, Warthmann, & Weigel, 2006). Three silencing constructs were generated according to http://wmd3.weigelworld.org/. The following sequences were targeted in *NtXIP1;1* (amiRNAI = TCGACTACGGTCACCATGAAA, amiRNAII = CCGCCGCGCTTGTCGGAATTA, amiRNAIII = ATGTTGGACACCATAGTGAT). The hairpin construct was amplified using the primers described in Table S1, and cloned downstream of the *En2pPMA4* promoter in pAUX3131 using KpnI and SacI. The expression cassette was then excised using I‐SceI and inserted into pPZP‐RCS2‐nptII. Only the silencing construct amiRNAII resulted in plants with a significant reduction in NtXIP1;1 protein levels.

### 2.5 Plant transformation

For *N. tabacum* transformation, the constructs were introduced into *Agrobacterium tumefaciens* (LBA4404 virGN54D; van der Fits, Deakin, Hoge, & Memelink, 2000) for subsequent leaf disk transformation (Horsch et al., 1986). *A. thaliana* was transformed using the above‐mentioned *A. tumefaciens* strain, harboring the respective construct, by floral dipping.

### 2.6 Antibody generation and western blotting

An antiserum was produced in rabbits against the amino‐terminal part of NtXIP1;1 using the synthetic peptide LGDEESQLSGGSNRVQ coupled to keyhole limpet hemocyanin carrier protein (Eurogentec). To test its specificity, the yeast strain BY4741 was transformed with either the empty vector pYeDP60u or vectors harboring one of the following coding sequences: *NtXIP1;1*α, *NtXIP1;1*β, *ZmNIP2;1*,* AtTIP2;1*,* ZmPIP1;2*,* ZmPIP2;5*. Transformed cells were grown on selective medium (2% glucose, 50 mM succinic acid/Tris base, pH 5.5, 0.7% yeast nitrogen base without amino acids [Difco], 2% agar‐agar). For protein extraction cells of pre‐cultures in liquid selective medium were transferred in Yeast Extract ‐ Peptone ‐ Galactose medium and grown until OD_600_ 0.8‐1. Cells were broken with acid washed glass beads in an extraction buffer [100 mM KH_2_PO_4_, pH 8, protease inhibitor (Roche, complete protease inhibitor tablets)] using a Precellys tissue homogenizer (Bertin). Microsomal fractions were isolated using centrifugation and resuspended in solubilization buffer (5 mM KH_2_PO_4_, 330 mM sucrose, 3 mM KCl, pH 7.8, protease inhibitors). Protein concentration was determined by Bradford (1976). Western blotting was performed using 15 μg of protein on a PVDF membrane. The anti‐NtXIP1;1 antiserum was used in a dilution of 1:1,000 and an alkaline phosphatase secondary antibody (Sigma) was used as a secondary antibody in a dilution of 1:5,000. Detection was performed with the NBT/BCIP Stock Solution (Roche).

### 2.7 Plant membrane protein extraction and western blotting

Fresh plant tissues were homogenized in 2 ml of extraction buffer (250 mM sorbitol, 60 mM Tris, 2 mM Na_2_EDTA, 10 mM dithiothreitol, pH 8 [HCl], protease inhibitors [Roche, complete protease inhibitor tablets]). Microsomal membrane fractions were isolated using centrifugation. The pellet was suspended in 5 mM KH_2_PO_4_, 330 mM sucrose, 3 mM KCl, pH 7.8 supplemented with protease inhibitors. Protein content was quantified using the Bradford method (Bradford, 1976). Western blotting was performed using 15–35 μg of protein on a PVDF membrane. The anti‐NtXIP1;1 antiserum was used in a dilution of 1:500 and an alkaline phosphatase secondary antibody (Sigma) was used as a secondary antibody in a dilution of 1:5,000. Detection was performed with the NBT/BCIP Stock Solution (Roche).

### 2.8 Rescue of boron deficiency phenotypes

*Nicotiana tabacum* NtXIP1;1 overexpression lines were fertilized weekly with nutrient solution MT (7.5 mM NH_4_NO_3_, 2.9 mM KH_2_PO_4_, 850 μM MgSO_4_, 340 μM K_2_SO_4_, 320 μM MnCl_2_, 52 μM CuSO_4_, 20 μM NaFeEDTA, 45 μM ZnSO_4_, 0.4 μM NaMoO_4_) plus 300 μM of boric acid. *Arabidopsis thaliana* overexpression lines of *NtXIP1;1* were grown on soil enriched up to a B content of 2.4 mg B/kg soil.

### 2.9 Boron‐deficient soil substrate growth conditions

The soil substrate was prepared according to Pommerrenig et al. (2018). In short: *N. tabacum* plants were germinated on soil substrate consisting of “Fruhstorfer Nullerde” supplemented with 0.5% CaCO_3_ and 0.3% CaO. The soil substrate was fertilized with nutrient solution MT (1 L/kg soil) with 0, 10, 200 μM (normal growth conditions) or 300 μM B(OH)_3_ (rescuing watering solution). Subsequently, the plants were watered with MQ water and once a week with 250 ml nutrient solution per kg soil without boric acid. No glassware was used in any process during the preparation of the nutrient solution or during the irrigation of the plants to avoid B contamination.

### 2.10 Boron toxicity soil substrate growth conditions

*Nicotiana tabacum* plants were germinated on soil substrate as described‐above supplemented with 200 μM of B(OH)_3_. After approximately 3 weeks, the plants were transferred to new pots. The soil was fertilized in four subsequent steps with the above‐described nutrient solution MT and either low, medium or high boric acid concentration to a final content of 2.4, 100 and 130 mg B/kg soil. Thereafter, plants were watered weekly with 250 ml nutrient solution per kg soil without boric acid.

### 2.11 HR‐ICP‐MS analysis

For elemental analysis, leaves, meristems, or stem material, epidermal peels and oocytes were dried at 65°C. Approximately 10 mg of plant dry matter or the oocytes was digested in nitric acid (HNO_3_) using a high‐performance microwave reactor (UltraClave IV; MLS GmbH). The elemental analysis was performed with a sector field high‐resolution ICP‐MS (Element 2, Thermo Fisher Scientific) using software v.3.1.2.242 (Eggert & von Wirén, 2013).

### 2.12 Xylem sap collection

*Nicotiana tabacum* wild type and NtXIP1;1 overexpressors were grown on soil until visible B deficiency symptoms occurred on the overexpressors. The stem was cut around 3 cm above the soil and a plastic tube was placed tightly around the cutting site. To avoid contaminations, the first 100 μl of xylem sap was discarded. Xylem sap was collected in the morning for 1–3 hr. The xylem sap was snap frozen in liquid nitrogen and stored at −80°C until further analysis using ICP‐MS.

### 2.13 Arabidopsis boron deficiency experiments in in vitro culture

To induce B deficiency, T2 plants were germinated on half‐strength MS medium (2.2 g/L MS salts with minimal organics [Duchefa], 1% sucrose, 0.7% Phytagel [Sigma], pH 5.8 [KOH]) containing the respective antibiotic. After 1 week, the plantlets were transferred to B deficiency medium (625 μM KH_2_PO_4_, 750 μM MgSO_4_, 1.5 mM CaCl_2_, 9.4 mM KNO_3_, 1 mM NH_4_NO_3_, 75 μM FeNa_2_EDTA, 0.055 μM CoCl_2_, 0.05 μM CuSO_4_, 50 μM MnSO_4_, 0.5 μM Na_2_MoO_4_, 15 μM ZnSO_4_, 2.5 μM KI, 1 mM MES, pH 5.5 [KOH], 1% Phytagel). The B deficiency medium was treated with MQ‐washed 3 g/L Amberlite IRA‐743 (Sigma) overnight prior to autoclaving. After 7 days of growth, roots were harvested for RNA extraction.

### 2.14 RNA extraction and RT‐PCR

RNA was extracted using the NucleoSpin^®^ RNA kit (Macherey‐Nagel) according to the manufacturers instructions. cDNA was synthesized using of 1 μg of RNA for subsequent RT‐PCR analysis.

### 2.15 Accession numbers

GenBank accession numbers: AtNIP5;1 (NP_192776.1), NtXIP1;1α (HM475295), NtXIP1;1β (HM475294).

## 3 RESULTS

### 3.1 Overexpression of NtXIP1;1 in *N. tabacum* results in abnormal growth

**Figure 1:**
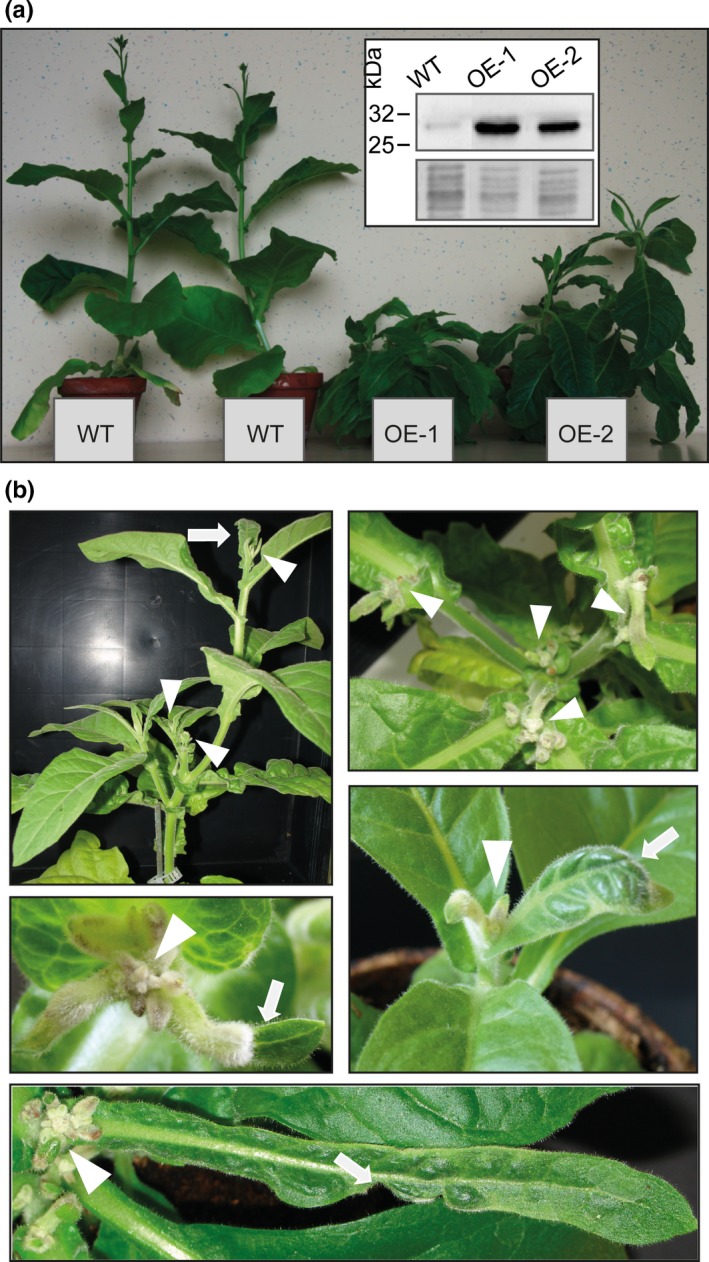
Boron deficiency symptoms of NtXIP1;1 overexpressing *N*. tabacum lines. (a) NtXIP1;1 overexpressing (OE‐1 and OE‐2) and wild type (WT) *N. tabacum* plants. The OE‐1 and OE‐2 are branched and have several shoot apical meristems. Inset: Western blot showing the NtXIP1;1 protein level in young leaves of 5‐week‐old soil grown WT, OE‐1 and OE‐2 lines detected using an anti‐NtXIP1;1 antibody (upper picture), and coomassie blue staining of the corresponding microsomal membrane fraction (lower picture). (b) Typical leaf tissue malformations and shoot apical meristem phenotypes observed in 5–6 week old tobacco lines overexpressing NtXIP1;1. White arrows highlight malformed leaves and white arrowheads meristems that stopped their development

Alteration of the endogenous expression level of a protein can give essential information about its physiological function *in planta*. We generated genetic constructs driving the expression of the *NtXIP1;1*α (hereafter referred to as *NtXIP1;1*) coding sequence under the control of constitutive overexpression promoters (i.e. either the *Nicotiana plumbaginifolia PMA4* promoter fused to two copies of the 35S enhancer (*En2pPMA4,* De Muynck et al., 2009) or the 35S Cauliflower Mosaic Virus [CaMV] promoter). To investigate NtXIP1;1 protein expression levels, an antibody was generated against the cytoplasmic N‐terminal part of the channel. Specificity of the antibody was tested using microsomal membrane fractions of *S. cerevisiae* expressing isoforms of different plant MIP subfamilies namely NtXIP1;1α, NtXIP1;1β, ZmNIP2;1, AtTIP2;1, ZmPIP1;2 or ZmPIP2;5. Only in protein extracts expressing either of the NtXIP1;1 splice variants, bands at approximately 30 and 55 kDa corresponding to the NtXIP1;1 monomer and dimer were detected (Figure S1). The detected signal is slightly lower than the expected molecular weight of the NtXIP1;1 monomer (34 kDa). The shift in molecular weight is frequently observed for the migration of membrane proteins and was previously observed for other MIP isoforms (Kobae, Mizutani, Segami, & Maeshima, 2006; Schüssler et al., 2008). Using the anti‐NtXIP1;1 antibody the T1 generation of *En2pPMA4*_*pro*_ *:NtXIP1;1* or *35S*_*pro*_ *:NtXIP1;1* tobacco transformants was screened for overexpression of NtXIP1;1. More than 30 independent lines showed elevated expression levels compared to wild type *N. tabacum* plants. Interestingly, most of these highly overexpressing lines showed abnormal growth approximately two and a half weeks after their transfer from the in vitro regeneration medium to soil. Young developing leaves were deformed, curled, puckered and dark green with abnormal vasculature patterns, the shoot apical meristem as well as subsequent forming meristems arrested their growth and died off. Resultant, multiple shoots with arrested meristem growth developed on one plant, leading to a branchy shoot phenotype. In case flowers succeeded in developing, they were infertile (Figure 1a,b) and no seeds were obtained. Strikingly, the appearance of the phenotype was associated with a high NtXIP1;1 expression level. Due to the incapability of the strong overexpression lines to set seeds and generate offspring, the transformation was repeated with identical results, namely plants which developed the above‐described severe symptoms. In the following, leaf‐tissue culture regeneration was performed to preserve transgenic lines. The independent OE‐1 and OE‐2 lines showing strong NtXIP1;1 overexpression under the control of the *En2pPMA4* promoter and the above‐described phenotypes were chosen for all subsequent investigations (Figure 1a,b).

### 3.2 Overexpression of NtXIP1;1 leads to boron deficiency in young tissues that can be remediated by a surplus boron fertilization

**Figure 2:**
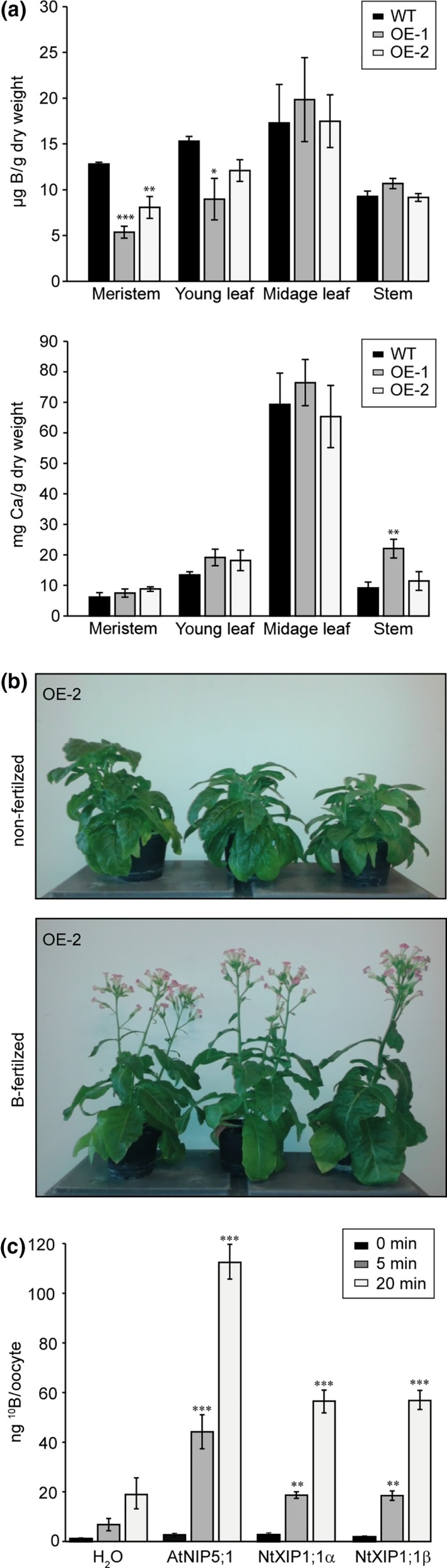
Boron and calcium concentrations in NtXIP1;1 overexpressing lines, rescue of the boron deficiency symptoms by boron surplus fertilization and facilitation of boric acid diffusion by NtXIP1;1 into *Xenopus* oocytes. (a) Boron (upper panel) and calcium (lower panel) concentrations in different tissues of 5‐week‐old wild type (WT) and NtXIP1;1 overexpressing OE‐1 and OE‐2 *N. tabacum* plant lines determined using ICP‐MS analysis. Chart bars represent means ± *SD* (*n* = 3 with leaf samples of five plants per biological replicate). Significant differences in plant B and Ca concentrations compared to the WT are indicated (*p < 0.05; **p < 0.01; ***p < 0.001 Student's *t* test). (b) Phenotypes of NtXIP1;1 overexpressing lines such as infertility, malformations of young leaves and shoot apical growth arrest (upper picture) can be overcome by additional B fertilization (lower picture). (c) ^10^Boron content in oocytes injected with water as a negative control, *AtNIP5;1* cRNA as a positive control, and *NtXIP1;1*α or *NtXIP1;1*β cRNA. Boron uptake rates were measured at the indicated time points after transfer to a bathing solution containing 5 mM ^10^B(OH)_3_ using ICP‐MS analysis. Chart bars represent means ± *SE* of 6 (0 and 5 min) or 10 (20 min) pools (*n* = 8–10 oocytes per pool) of oocytes from two independent experiments. Asterisks indicate significant different ^10^B levels between the water‐injected negative control oocytes and the oocytes heterologously expressing the indicated aquaporin isoform (**p < 0.01; ***p < 0.001; Student's *t* test) [Colour figure can be viewed at wileyonlinelibrary.com]

The phenotype of the NtXIP1;1 overexpressing lines was striking concerning the malformed leaves, the dying meristems and the infertility. The symptoms resemble typical characteristics of either a calcium (Ca) or a B deficiency phenotype. Long‐distance transport of both B and Ca in plants follows and depends on the transpiration stream. Leaves, meristems and stems of wild type and NtXIP1;1 OE‐1 and OE‐2 lines were analyzed using ICP‐MS analysis for their B and Ca concentrations. While the B concentration was significantly lower in symptomatic young leaves and in meristems of the overexpressing lines, the Ca concentration was not altered pointing towards a B deficiency‐related phenotype (Figure 2a). We deduced that xylem‐mediated transport fluxes to the meristem and young leaves were not impaired in the overexpressing lines as otherwise a lower Ca concentration would have been measured.

Deficiency of a nutrient can presumably be remedied by supplying a surplus of the respective element. Wild type and NtXIP1;1 overexpressing lines were grown on soil for 10 days before being transferred to individual pots. From this point on, the pots were watered with a nutrient solution containing 300 μM of B. The overexpressing lines were thus able to generate fertile flowers and to set seeds (Figure 2b). With this additional B supply, it was possible to obtain the T2 and following generations of the transgenic plants. These data demonstrated that the NtXIP1;1 overexpression phenotypes result from B deficiency in young leaves and meristems, which can be overcome by supplying additional B. From there on, the seeds obtained from the T3 generation were used in the subsequent experiments.

### 3.3 NtXIP1;1 is a boric acid channel

We previously showed that NtXIP1;1 might facilitate the transport of boric acid, using indirect toxicity growth assays in *S. cerevisiae* cells (Bienert et al., 2011). To demonstrate the ability of NtXIP1;1 to channel boric acid in a direct and quantitative uptake assay, NtXIP1;1 was expressed in *X. laevis* oocytes and incubated in a solution containing 5 mM of ^10^Boric acid. Boron uptake rates into the oocytes were measured for different time periods and B concentrations were determined via ICP‐MS analysis. As seen for the water‐injected negative control oocytes, boric acid can freely diffuse across membranes over time (Figure 2c). However, expression of the known B channel AtNIP5;1 increased the inward flow of boric acid significantly. Similarly, expression of the two splice variants NtXIP1;1α and NtXIP1;1β increased the uptake of boric acid into oocytes significantly compared to the negative control oocytes (Figure 2c), demonstrating that NtXIP1;1 is a functional boric acid channel.

### 3.4 Silencing of *NtXIP1;1* in *N. tabacum* does not lead to an increased sensitivity to boron deficiency nor toxicity, neither to a variation in boron tissue concentration

**Figure 3:**
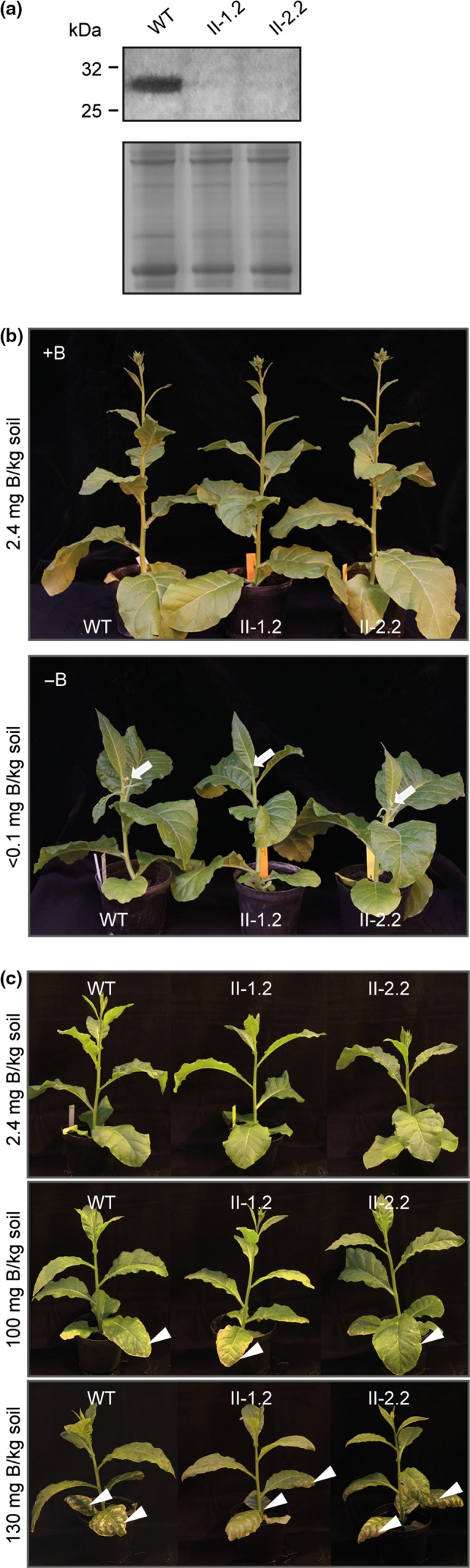
Characterization of *N. tabacum* plants silenced in NtXIP1;1 expression. (a) Western blotting of whole leaf microsomal fractions of 2‐week‐old plantlets of *N. tabacum* wild type (WT) and amiRNA *NtXIP1;1*‐silenced lines II‐1.2 and II‐2.2 using the NtXIP1;1‐antibody demonstrating efficient silencing of NtXIP1;1 protein in the amiRNA lines (upper panel). SDS‐gel loading controls are displayed showing the coomassie blue staining of the corresponding microsomal fractions (lower panel). (b) *N. tabacum* WT and *NtXIP1;1*‐silenced lines II‐1.2 and II‐2.2 grown on B‐sufficient (+B, 2.4 mg B/kg soil) and B‐deficient soil substrate (−B, <0.1 mg B/kg soil). The chlorotic shoot apical meristems, which have arrested their growth due to the B‐deficient conditions are marked by a white arrow. (c) *N. tabacum* WT and *NtXIP1;1*‐silenced lines II‐1.2 and II‐2.2 grown on B‐sufficient (2.4 mg B/kg soil) and toxic B (100 and 130 mg B/kg soil) soil substrate concentrations. White arrowheads indicate typical B‐toxicity induced necrotic regions in leaf tips and margins of oldest leaves (100 mg B/kg soil) and also the gradual extensions of the necrotic regions along the leaf blade and in younger leaves under severe toxic B conditions (130 mg B/kg soil)

In order to further decipher the physiological role of NtXIP1;1 in *N. tabacum* in more detail, silencing of *NtXIP1;1* was targeted using the artificial micro RNA technique (amiRNA) (Schwab et al., 2006). Three different amiRNA constructs were generated of which one resulted in an efficient *NtXIP1;1* silencing. Two independent lines showing efficient silencing on the protein level were chosen for further investigations (Figure 3a). The amiRNA lines did not show any obvious phenotype compared to wild type plants. Challenging them with B‐deficient conditions induced B deficiency symptoms such as deformations of young leaves and shoot apical meristem growth retardation as observed with wild type plants (Figure 3b). Similarly, toxic B conditions led to severe and typical B toxicity symptoms such as necrotic regions in leaf tips and margins, first in oldest leaves and subsequently in younger leaves, which gradually extend throughout the leaf blades on both, wild type and *NtXIP1;1* silenced lines without any difference in growth height (Figure 3c).

**Figure 4:**
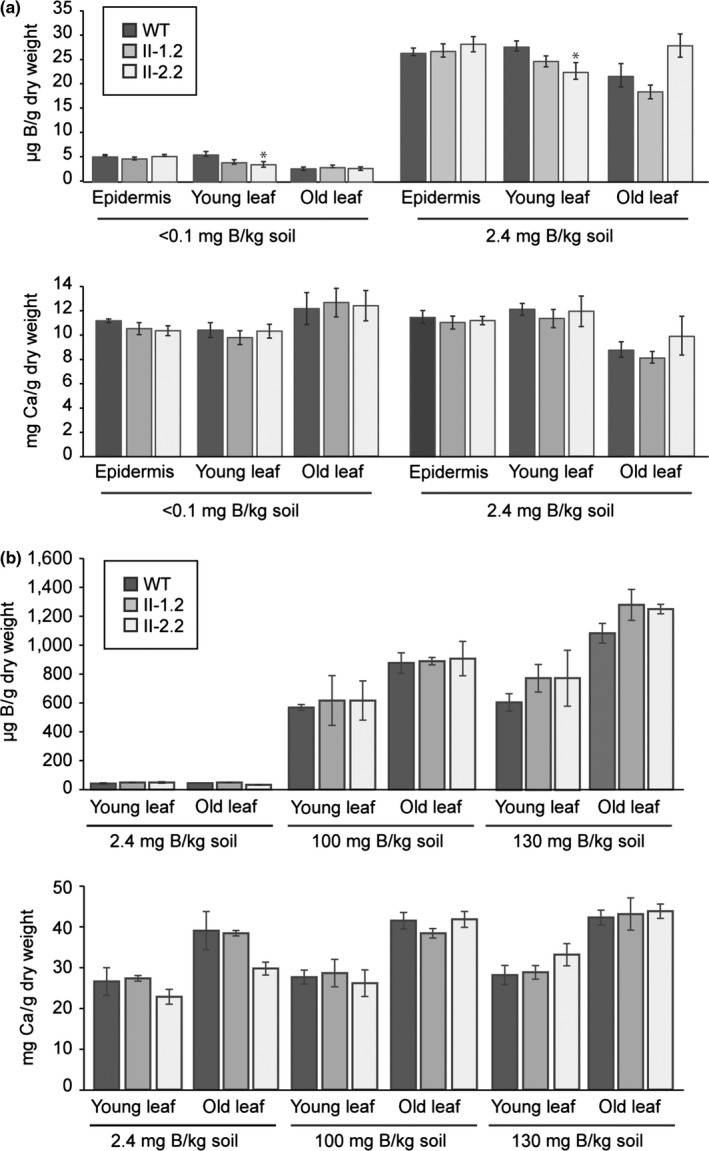
Boron and calcium concentrations of wild type and *NtXIP1;1*‐silenced *N. tabacum* leaf samples grown under toxic or deficient soil boron concentrations. (a) Boron (upper panel) and calcium (lower panel) concentrations of abaxial epidermal peels, young (6th leaf) and old (3rd leaf) leaf samples of wild type (WT) and *NtXIP1;1*‐silenced plants II‐1.2 and II‐2.2 grown in B‐sufficient (2.4 mg B/kg soil) or B‐deficient (<0.1 mg B/kg soil) soil substrate. Values represent means ± *SE* (*n* = 6). Asterisks indicate significant different nutrient concentrations between the WT and the *NtXIP1;1*‐silenced plants (*p < 0.05; Student's *t* test). (b) Boron (upper panel) and calcium (lower panel) concentrations of young (6th leaf) and old (3rd leaf) non‐necrotic leaf samples of WT and *NtXIP1;1*‐silenced plants grown on B‐sufficient (2.4 mg B/kg soil) or toxic B (100 or 130 mg B/kg soil) soil substrate concentrations. Values represent means ± *SE* (*n* = 3)

Boron concentrations in plant tissue can drop quite substantially without resulting in an obvious phenotype. Therefore, the tissue B concentration of wild type and *NtXIP1;1*‐silenced lines might differ due to the absence of NtXIP1;1 in certain tissues to a level above the deficiency threshold. As *NtXIP1;1* is dominantly expressed in the leaf epidermis and sub‐epidermal cells in above ground tissues (Bienert et al., 2011), abaxial epidermal strips as well as whole leaf samples were harvested from plants grown under standard and B‐deficient or excess conditions and subjected to an element analysis. Under B deficiency, the tissue B concentration decreased substantially in both the wild type and the *NtXIP1;1*‐silenced lines. However, neither in the epidermis nor in the whole leaf tissue did the B concentration differ between the lines (Figure 4a). Similarly, growth on soil with a high B content led to an increased tissue B concentration to a comparable level in wild type and mutant plants (Figure 4b). To assess whether the xylem‐mediated long‐distance transport of *NtXIP1;1*‐silenced plants is potentially altered under B‐deficient or B‐toxic conditions in comparison to the wild type, Ca concentrations were analyzed in these plants. Similar to B concentrations, no significant changes in Ca concentrations were found (Figure 4a,b).

### 3.5 NtXIP1;1 expression responds to the boron nutritional status

**Figure 5:**
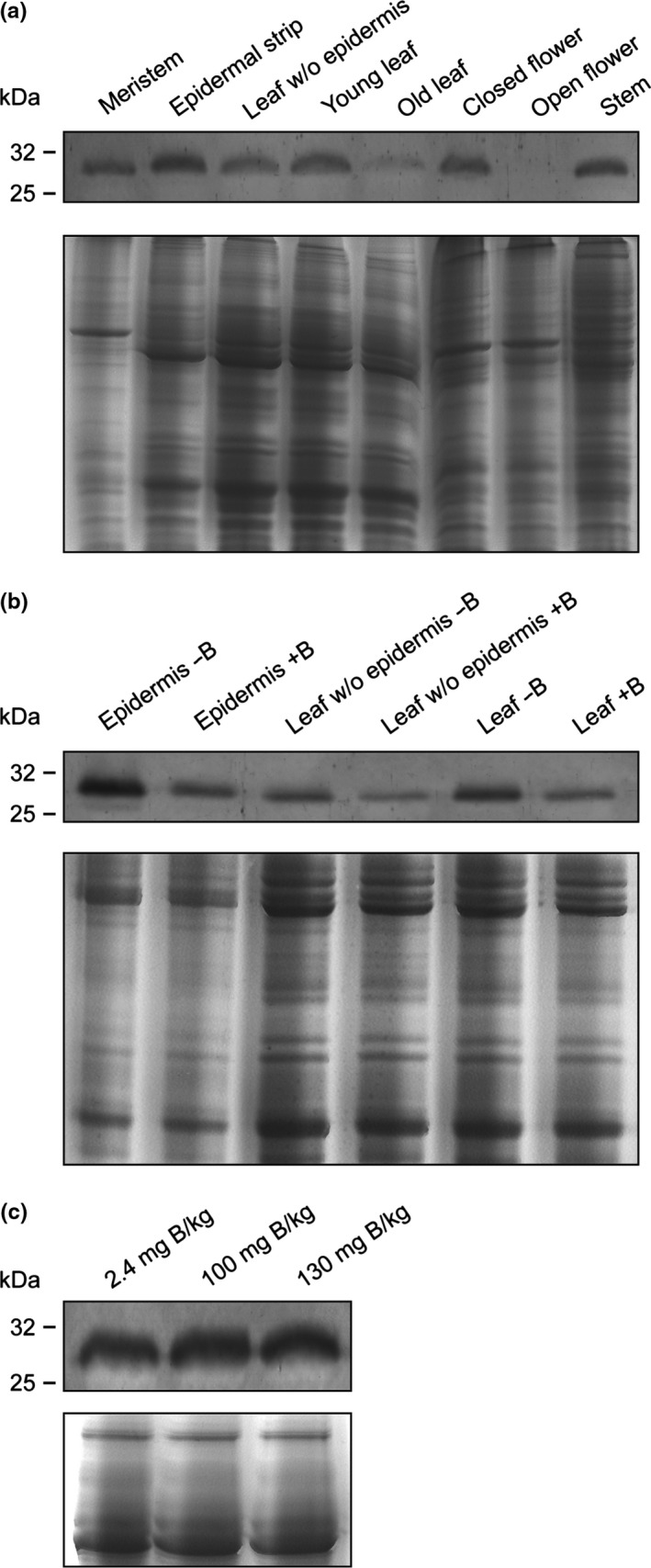
NtXIP1;1 protein levels in *N. tabacum* tissues in response to the boron (B) nutritional status. (a) Western blot showing the NtXIP1;1 protein levels in microsomal fractions of diverse *N. tabacum* tissues using the anti‐NtXIP1;1 antibody (upper panel) and coomassie blue staining of a SDS‐PAGE gel containing the corresponding microsomal fractions, used as a loading control (lower panel). (b) Western blot showing the NtXIP1;1 protein levels in abaxial epidermal strips, leaf without abaxial epidermis and whole leaf samples of wild type *N. tabacum* plants grown in B‐sufficient (+B, 2.4 mg B/kg soil) or B‐deficient (−B, <0.1 mg B/kg soil) soil substrate (upper panel) and coomassie blue staining of a SDS‐PAGE gel containing the corresponding microsomal fractions, used as a loading control (lower panel). (c) Western blot showing the NtXIP1;1 protein levels in microsomal fractions prepared from wild type *N. tabacum* plants grown under B‐sufficient (2.4 mg B/kg soil), B excess (100 mg B/kg soil) or toxic B (130 mg B/kg soil) soil concentrations (upper panel). The loading control is displayed in the lower panels showing a coomassie blue staining of a SDS‐PAGE gel containing the corresponding microsomal fractions

To investigate whether the expression of NtXIP1;1 is responsive to the B nutritional status of wild type plants, its expression profile was investigated in tobacco plants. Microsomal fractions were isolated from various tissues and expression was investigated using Western blotting. NtXIP1;1 was expressed in the root (Figure S2), leaves, stem, and flowers (Figure 5a). Interestingly, higher NtXIP1;1 protein levels could be detected in developmentally younger organs compared to the corresponding older organs (e.g. young vs. old leaves and closed vs. open flowers) which points towards a physiological function in young and growing tissues. As the *NtXIP1;1* promoter activity was high in the leaf epidermis, as visualized using the GUS reporter gene expression (Bienert et al., 2011), we studied the protein level in epidermal peels. NtXIP1;1 protein levels correlated with previously observed promoter activity levels (Bienert et al., 2011) and were higher in epidermal peels than in leaves lacking the abaxial epidermis.

In order to study the effect of B deprivation or toxicity on the protein level of the B‐permeable NtXIP1;1, wild type tobacco plants were grown on low (<0.1 mg B/kg soil), adequate (2.4 mg B/kg soil) and toxic (100 and 130 mg B/kg soil) B concentrations. Boron deficiency and toxicity were confirmed by the occurrence of typical visible symptoms (Figure 3b,c, plants on the left). To assess NtXIP1;1 protein levels under B‐deficient conditions, microsomal fractions of abaxial epidermal strips, leaves without abaxial epidermis and whole leaf tissue were harvested at the onset of visible B deficiency symptoms. NtXIP1;1 protein levels were higher throughout all leaf tissue samples upon B deprivation (Figure 5b). Under toxic B conditions, NtXIP1;1 protein levels were not altered indicating that NtXIP1;1 protein expression is only responsive to B deficiency (Figure 5c).

### 3.6 Deregulated expression of NtXIP1;1 reduces the B concentration in the xylem sap

**Figure 6:**
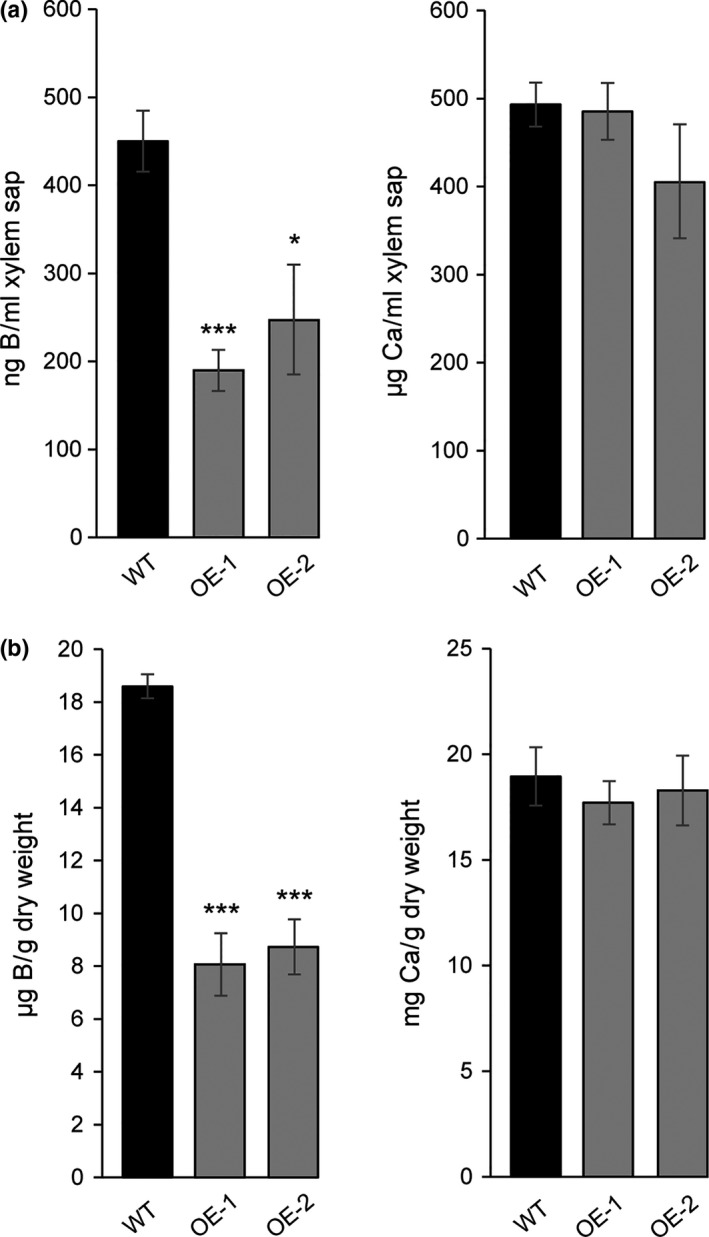
Boron and calcium concentrations in the xylem sap of wild type and NtXIP1;1 overexpressing lines grown on fully fertilized and boron‐deficient soil. (a) B and Ca xylem sap concentrations of wild type (WT) or NtXIP1;1 OE‐1 and ‐2 lines grown on soil. Bar charts represent means ± *SE* (*n* = 3–8). Significant differences in the respective element concentration compared to WT are marked with asterisk (*p < 0.05; ***p < 0.001; Students *t* test). (b) B and Ca concentration of the youngest developed leaf of WT or NtXIP1;1 OE‐1 and ‐2 line plants used in (a)

Boron is translocated from roots to the above ground tissue along the transpiration stream via the xylem sap. The B concentration in the xylem sap of WT and NtXIP1;1 overexpressors was determined in order to investigate whether the delivery of B to the shoot, and subsequently to the lowly transpiring young leaves and meristem sink tissues is hampered due to the overexpression of NtXIP1;1 and therewith to mechanistically explain the phenotype of the overexpressors. Xylem sap was collected from 8‐week‐old plants grown on soil when visible B deficiency symtpoms occurred. The shoot was dissected in the morning and silicon tubes were gently imposed on the remaining stem. Xylem sap was collected for 1–3 hr. ICP‐MS analysis showed that the B concentration in the overexpressors is significantly lower compared to that of wild type plants implying that the decreased B xylem concentration restricts the amount of B reaching the low‐transpiring sink tissues (Figure 6a). On the contrary, xylem sap Ca concentrations did not differ between the different lines demonstrating that the xylem transport as such is not impaired. B deficiency in the NtXIP1;1 overexpressors was confirmed by measuring the B concentration in the youngest developed leaf using ICP‐MS analysis. B concentrations in the NtXIP1;1 overexpressors was significantly lower in both lines compared to the WT (Figure 6b).

### 3.7 Overexpression of NtXIP1;1 in Arabidopsis results in abnormal growth in an expression level‐dependent manner

**Figure 7:**
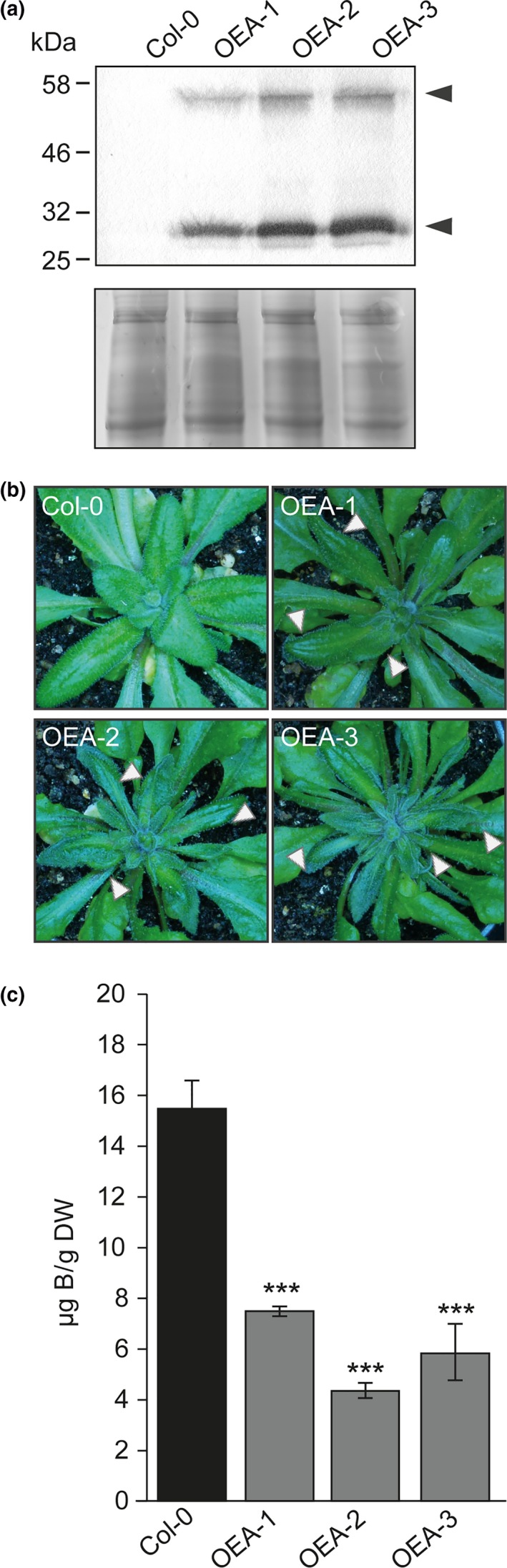
Phenotype and boron tissue concentration of Arabidopsis plants overexpressing NtXIP1;1. (a) Western blot of microsomal fractions of wild type Arabidopsis (Col‐0) and NtXIP1;1 overexpressing lines (OEA‐1 to ‐3) using the anti‐NtXIP1;1 antibody (upper panel). The monomeric and dimeric NtXIP1;1 forms are marked (black arrowheads). Coomassie staining of a SDS‐PAGE gel containing corresponding microsomal fractions, used as a loading control (lower panel). (b) Boron deficiency symptoms in young leaves of Arabidopsis lines overexpressing NtXIP1;1 (OEA‐1 to ‐3) in comparison to the Col‐0 wild type plant when grown on fully fertilized standard greenhouse soil substrate. Curled‐down and malformed B deficiency symptoms displaying young leaves are marked with white arrowheads. (c) Boron concentration of leaf samples of wild type Col‐0 Arabidopsis (black bar) and NtXIP1;1 overexpressing lines (OEA‐1 to ‐3) (gray bars) (of [b]) determined using ICP‐MS analysis. Asterisks indicate significant different B concentrations between the WT and the NtXIP1;1‐overexpressor lines (***p < 0.001; Student's *t* test). Values represent means ± *SE* (*n* = 4)

Arabidopsis belongs to the *Brassicaceae* family, which does not encode XIPs (Danielson & Johanson, 2008). Aiming to investigate the B deficiency phenotype linked to the overexpression of NtXIP1;1 in an independent plant species, we overexpressed NtXIP1;1 in Arabidopsis using the *35S CaMV* promoter. The T1 generation was screened for positive overexpression lines using Western blotting. Interestingly, lines showing a strong NtXIP1;1 overexpression developed curled, cupped‐down, dark green rosette leaves at a later stage of vegetative growth and were unable to develop fertile flowers (Figure 7a,b). These symptoms resemble the phenotype seen in NtXIP1;1 overexpressing *N. tabacum* plants. Young leaves were subsequently harvested and analyzed for their B and Ca concentration using ICP‐MS elemental analysis. While the B concentration significantly decreased in those lines displaying a phenotype, the Ca concentration was not altered in a phenotype‐dependent manner (Figures 7c and S3). Interestingly, the phenotype could be rescued by increasing the soil B content to 2.4 mg B/kg soil (Figure S4) confirming a B deficiency‐related phenotype in Arabidopsis comparable to the phenotype observed by overexpression of NtXIP1;1 in its endogenous plant background.

### 3.8 NtXIP1;1 rescues the boron deficiency phenotype of *Atnip5;1*

**Figure 8:**
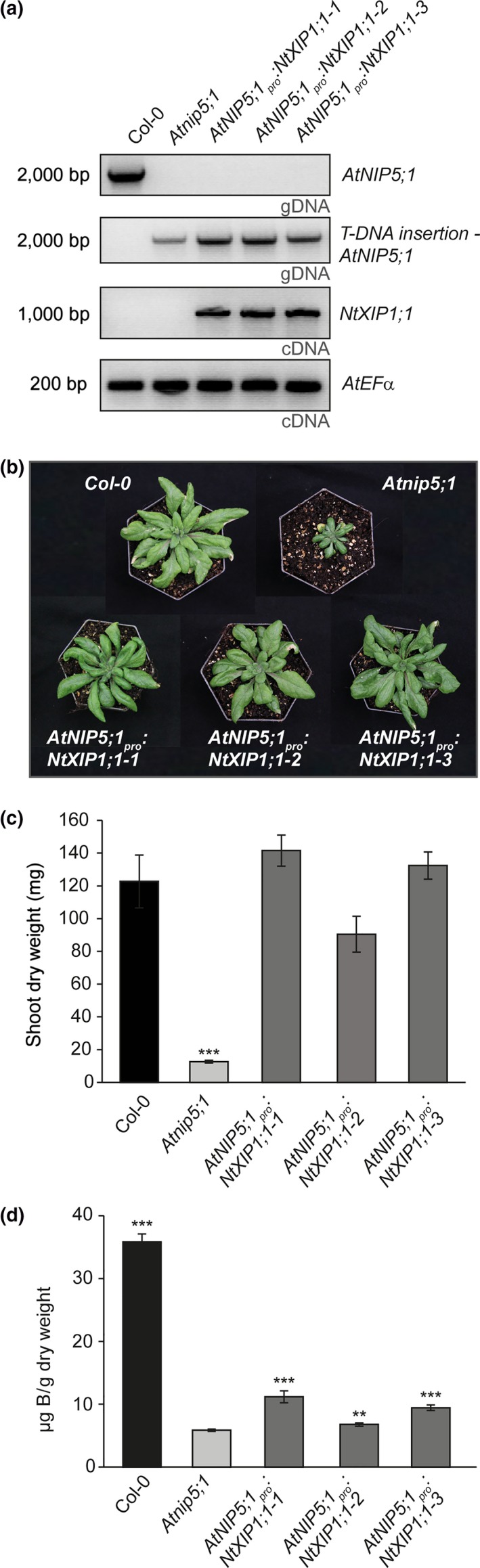
Partial growth complementation of the Arabidopsis *Atnip5;1* knockout plants by tissue‐specific expression of *NtXIP1;1*. (a) PCR and RT‐PCR on leaf genomic DNA (gDNA) and root RNA, respectively, extracted of Arabidopsis wild type (Col‐0) or *Atnip5;1* knockout plants and *Atnip5;1* knockout lines transformed with the *AtNIP5;1*_*pro*_ *:NtXIP1;1* construct, grown under B‐deficient in vitro growth conditions. PCR detected the genomic region of *AtNIP5;1* or the T‐DNA insertion in the *AtNIP5;1* genomic sequence. RT‐PCR detected *NtXIP1;1* cDNA and the Elongation factor α (*EF*α) as a reference. (b) Partial rescue of the B deficiency phenotype of the *Atnip5;1* knockout plants growing on standard soil substrate by the expression of *NtXIP1;1* under the control of the *AtNIP5;1* promoter in comparison to the wild type (Col‐0) and *Atnip5;1* knockout plants. (c) Shoot dry weight of wild type (Col‐0) or *Atnip5;1* knockout plants and independent *Atnip5;1* knockout lines expressing the *AtNIP5;1*_*pro*_ *:NtXIP1;1* construct. Bar charts represent means ± *SD* (*n* = 4). Significant differences in the shoot dry weight compared to Col‐0 are marked with an asterisk (***p < 0.001; Student's *t* test). (d) Leaf B concentration of wild type (Col‐0) or *Atnip5;1* knockout plants and independent *Atnip5;1* knockout lines expressing with the *AtNIP5;1*_*pro*_ *:NtXIP1;1* construct. Bar charts represent means ± *SD* (*n* = 4). Significant differences in leaf B concentrations compared to *Atnip5;1* knockout plants are assessed (**p < 0.01; **p < 0.001; Student's *t* test)

To demonstrate unequivocally that NtXIP1;1 is a functional boric acid channel in plants, we expressed NtXIP1;1 under the control of the *AtNIP5;1* promoter sequence in the *Atnip5;1* knockout background (Takano et al., 2006). *Atnip5;1* knockout plants suffer substantially from B deficiency and need additional B in order to develop normally under standard growth conditions (Figure S4). Independent lines transformed with an *AtNIP5;1*_*pro*_:*NtXIP1;1* construct were grown on B‐deficient growth media and selected for *NtXIP1;1* expression using RT‐PCR on root samples, where *AtNIP5;1* is predominantly expressed under B‐deficient conditions (Figure 8a). *NtXIP1;1* expressing lines grew significantly better than *Atnip5;1* knockout plants although they still displayed weak B deficiency symptoms throughout their life cycle when grown under standard soil growth conditions (Figure 8b). Analyzing the shoot dry weight of the individual T3 lines showed that the shoot dry weight reached wild type levels (Figure 8c). Measuring the tissue B concentration of the NtXIP1;1‐complemented *Atnip5;1* lines indicated that it was still in the range of B deficiency, but was significantly increased compared to the *Atnip5;1* knockout line (Figure 8d). The increased B uptake capacity by the expression of *NtXIP1;1* substantially improved plant performance under identical growth conditions though plants could still not reach maturity without additional B supply.

## 4 DISCUSSION

X Intrinsic Proteins are one of the most recently described aquaporin subfamilies and, therefore, comparatively little knowledge on their physiological impact on plant performance is available. After their discovery and the observation that this subfamily was lost in monocots and in some dicots such as the *Brassicacea* (Danielson & Johanson, 2008), XIP isoforms have been identified and their expression profile under different growth conditions was studied in various plant species (Giovannetti et al., 2012; Lopez et al., 2016; Noronha et al., 2016). Heterologous expression analysis revealed their ability to facilitate the diffusion of small solutes such as glycerol, hydrogen peroxide, urea or boric acid but no or only marginal water permeability (Ampah‐Korsah et al., 2016; Bienert et al., 2011; Giovannetti et al., 2012; Lopez et al., 2016). So far, XIP‐mediated boric acid permeability was only deduced based on XIP‐expressing yeast growth inhibition experiments (Ampah‐Korsah et al., 2016; Bienert et al., 2011; Noronha et al., 2016). This approach allows only an indirect assessment of the B transport activity. Here, we demonstrated that NtXIP1;1 conducts boric acid in direct and qualitative uptake measurements using oocytes (Figure 2c).

We then investigated the physiological role and possible impact of NtXIP1;1 on B transport *in planta*. Surprisingly, overexpression of NtXIP1;1 in *N. tabacum* led to an abnormal growth phenotype reflected in malformation of young leaves, growth arrest of the shoot apical meristem as well as subsequent forming meristems and infertility (Figure 1), symptoms linked to B deficiency. Similar symptoms were observed when NtXIP1;1 was overexpressed in *A. thaliana,* a species that lacks any XIP gene in its genome (Figure 7b). Elemental analysis of the B and Ca concentration in the young, malformed leaves and meristems revealed that the NtXIP1,1 overexpressing tobacco and Arabidopsis lines had significant lower B concentrations than the wild type while the Ca concentration was unvaried (Figures 2a, 8c, S3). To that effect, fertilizing the overexpressor lines in both species with a surplus of B fully restored plant growth and fertility and prevented malformations of leaves (Figures 2b, S4a) showing that overexpression of NtXIP1;1 in its endogenous but also in a heterologous plant background leads to B deficiency in young leaves. Interestingly, a similar observation i.e. a nutrient deficiency phenotype due to a nutrient transporter overexpression was made when overexpressing the Ca^2+^/H^+^ antiporter *AtCAX1* in *N. tabacum* (Hirschi, 1999). Upon overall expression of this naturally Ca‐induced gene product, tobacco exhibits severe Ca deficiency symptoms in the shoot apical meristem and young leaves and a concomitant low intrinsic Ca concentration in the corresponding tissues (Hirschi, 1999). Calcium surplus fertilization circumvents the Ca deficiency phenotypes (Hirschi, 1999). Timely expression of CAX1 is concluded to be essential for maintaining Ca homeostasis. Similarly, plants that overexpress *AtGluR2*, which represents a channel that unloads Ca from the xylem, show impaired Ca utilization and display Ca deficiency symptoms, such as browning and death of the shoot apex, necrosis of leaf tips, and deformation of leaves, which were alleviated by Ca surplus treatment (Kim, Kwak, Jae, Wang, & Nam, 2001). An analog incidence of nutrient deficiency symptoms in plants overexpressing Ca and B transporting proteins is interesting, as these two nutrients share characteristic properties with respect to plant nutritional aspects (Marschner, 2011). Calcium and B are the two nutrients whose translocation and distribution within the plant is highly dependent on the transpiration stream along the vasculature and which therefore display similar deficiency phenotypes mainly in young not yet fully developed and low‐transpiring tissues (Marschner, 2011). Moreover, both elements share essential molecular functions within the primary cell wall of plants by crosslinking sugars in the pectin fraction (Marschner, 2011). Our observations that constitutive overexpression of a transporter of a xylem‐mobile nutrient resulted in severe nutrient deficiency symptoms in young growing tissues are therefore in agreement with previous findings of other studies. Furthermore, we investigated the B concentration in the xylem sap of wild type as well as NtXIP1;1 overexpressors. Xylem B concentrations were significantly lower in the overexpressors demonstrating that the shoot‐driven B translocation is hampered in the overexpressers which is the limiting element for adequate delivery of sufficient B to low‐transpiring tissues like the young and developing leaves as well as the meristem (Figure 6). In respect to B translocation from the soil to the shoot it was shown that both the AtNIP5;1 boric acid channel as well as the active xylem loading transporter AtBOR1 have to be localized polarly in specific root cell types to maintain the important directional nutrient transport of B (Takano, Miwa, Yuan, von Wirén, & Fujiwara, 2005; Wang et al., 2017). Recently it was speculated, that improper localization of AtNIP5;1 at the stele side of the cells can impair the B gradient from the cytosol to the apoplast generated by AtBOR1 (Yoshinari & Takano, 2017). Ubiquitous overexpression of the physiologically active B channel NtXIP1;1 in roots will accordingly disturb local B gradients, important for generating the directional flow of B towards the shoot.

We showed that NtXIP1;1 was more strongly expressed in young and developing leaves and flowers compared to mature leaves and flowers (Figure 5a), suggesting that NtXIP1;1 endogenously functions in newly forming tissues in which cells are still elongating and cell walls have not yet reached maturity. Remarkably, NtXIP1;1 protein level was enriched in the leaf epidermis compared to leaf tissue lacking the abaxial epidermis (Figure 5a,b). Epidermal and sub‐epidermal cells and especially their walls potentially demand specific properties during cell growth due to their peripheral surface‐level location and protective function for plants. We hypothesize that the B status of these cells and their walls is of eminent importance and has to be tightly controlled. Surprisingly, the molecular and chemical knowledge on epidermal primary cell wall properties is scarce and almost absent. Our data suggest that XIP1;1 functioning contributes to the B partitioning in these structures.

In rice and Arabidopsis, the boric acid channels OsNIP3;1 and AtNIP6;1 are important for B allocation into young and developing tissues and its distribution amongst shoot tissues (Hanaoka, Uraguchi, Takano, Tanaka, & Fujiwara, 2014; Tanaka, Wallace, Takano, Roberts, & Fujiwara, 2008). Both, *OsNIP3;1* and *AtNIP6;1* RNAi or knockout plants show disturbed B distribution patterns leading to B deficiency phenotypes and both genes are expressed around the vascular bundles and the xylem in leaf tissues complying the requirements to function in B logistics within and between shoot tissues (Hanaoka et al., 2014; Tanaka et al., 2008). NtXIP1;1 is not expressed in the vascular bundle whereas expression is dominant in cell layers facing the environment in above ground tissues which implies a physiological function different from the long‐distance distribution via the vasculature. This can also explain the lacking phenotype of the NtXIP1;1‐silenced plants at the whole plant, organ and tissue level. Very likely, NtXIP1;1 plays a role in the distribution of B between the cytoplasm and the apoplast which has to be fine‐adjusted especially in the root as well as in young and growing epidermal and parenchymal cells which have to withstand dynamic pressure challenges during growth. Such a function of NtXIP1;1 is further substantiated by the fact that leaf tissue B concentration of the *NtXIP1;1*‐silenced lines and the wild type grown on deficient and toxic B soil concentrations was not significantly different (Figure 4). Most commonly, MIP subfamilies are composed of a few closely related and functionally redundant isoforms, which makes the identification of the function of individual channels difficult and tricky. Obvious morphological or physiological phenotypes at the whole plant level are sometimes even absent in multiple *MIP* gene knockouts (Martre et al., 2002; Schüssler et al., 2008). This fact also seems to hold true for XIPs. Based on expressed sequence tag data and previous cloning approaches (Bienert et al., 2011) it is evident that the allotetraploid genome of *N. tabacum* encodes for multiple *XIP* genes. This suggests that the biological impact of a single isoform on a specific physiological function is likely masked by the functional redundancy of the XIP family in tobacco. Therefore, we propose that a missing phenotype of the amiRNA *NtXIP1;1*‐silenced plants is also further due to a functional redundancy with other XIP isoforms and/or other B transport proteins such as NIPs or BORs. Alternatively, the silencing of *NtXIP1;1* may be imperfect and not 100% complete in the amiRNA lines although no XIP1;1 has been detected using Western blotting (Figures 3). Marginal XIP1;1 channel protein translation may remain within key tissues which may be sufficient to fulfill its physiological B transport function and repress the development of a detectable phenotype.

Considering that either functional redundancy or an imperfect silencing is causing the lack of phenotypes, a role of NtXIP1;1 in the extrusion or redistribution at the periphery of the stem or young leaves, or the subcellular partitioning between the cytoplasm and the apoplast is anyhow possible and will have to be investigated in future.

AtNIP5;1 is a boric acid channel of essential physiological importance for B uptake into *A. thaliana*. The *Atnip5;1* knockout line is severely hampered in development due to a low B uptake capacity (Takano et al., 2006). *AtNIP5;1* promoter activity is induced by B deficiency and drives expression specifically in the root cap, the epidermis and endodermis (Tanaka et al., 2016; Wang et al., 2017). We used the promoter sequence of *AtNIP5;1* to express *NtXIP1;1* in the *Atnip5;1* knockout background and obtained individual lines expressing *NtXIP1;1* in the root under B‐deficient growth conditions (Figure 8a). The *Atnip5;1* knockout plants suffer from severe B deficiency when grown under standard growth conditions (Figure 8b, S4). Interestingly, expression of *NtXIP1;1* under the control of the *AtNIP5;1* promoter complemented the plant growth phenotype compared to the *Atnip5;1* mutant (Figure 8b,c). The *AtNIP5;1*_*pro*_:*NtXIP1;1* lines have a higher B leaf tissue concentration compared to *Atnip5;1* knockout plants though they do not reach wild type levels (Figure 8d). AtNIP5;1 is polarly localized in the root exo‐ and endodermis towards the soil side of the root (Wang et al., 2017). This polar localization is indispensable for efficient translocation of B towards the shoot (Wang et al., 2017). Most likely NtXIP1;1 expressed in place of AtNIP5;1 does not localize in an identical manner to the distal side of the cell and, therefore, cannot fully replace AtNIP5;1 function to restore wild type B uptake levels. However, the increase in B concentration in the leaves of the *AtNIP5;1*_*pro*_:*NtXIP1;1* lines demonstrated that NtXIP1;1 expressed in the root enhances B uptake and, therefore, is a functional boric acid channel in *planta*.

In conclusion, overexpression of NtXIP1;1 leads to an abnormal organ‐ and cell‐type specific expression which disturbs correct B distribution throughout *N. tabacum* and *A. thaliana* leading to a B deficiency phenotype. In *A. thaliana* NtXIP1;1 is able to complement the *Atnip5;1* knockout phenotype proving its ability to function as a B channel in plants. Wild type tobacco plants up‐regulate NtXIP1;1 under B‐deficient conditions in young tissues which depend on a high and steady B supply for their growth and tissue function. This highlights the contribution of NtXIP1;1 to the regulation of the plants B nutritional status. Hence, coordinated expression of boric acid channels, such as NtXIP1;1, as well as cell‐ and tissue‐specific expression is indispensable for accurate B distribution within the plant.

## CONFLICT OF INTEREST

The authors declare no conflict of interest.

## AUTHOR CONTRIBUTIONS

G.P.B. and F.C. conceived the project, supervised this study and serve as the authors responsible for contact and ensure communication. M.D.B., B.M., G.P.B., and F.C. designed the research. M.B.D., B.M., D.C., and G.P.B. performed the research. M.D.B., B.M., D.C., G.P.B., and F.C. analyzed the data. M.D.B. and G.P.B. drafted the article. All of the authors discussed the results and commented on the manuscript.
