# Supplementary material for: Overexpression of X Intrinsic Protein 1;1 in Nicotiana tabacum and Arabidopsis reduces boron allocation to shoot sink tissues
Source: Plant Direct. 2019 Jun 5;3(6):e00143. doi: 10.1002/pld3.143 (PMC6549384; doi:10.1002/pld3.143)
Supplement: Supplementary file 1 [file PLD3-3-e00143-s001.pdf]

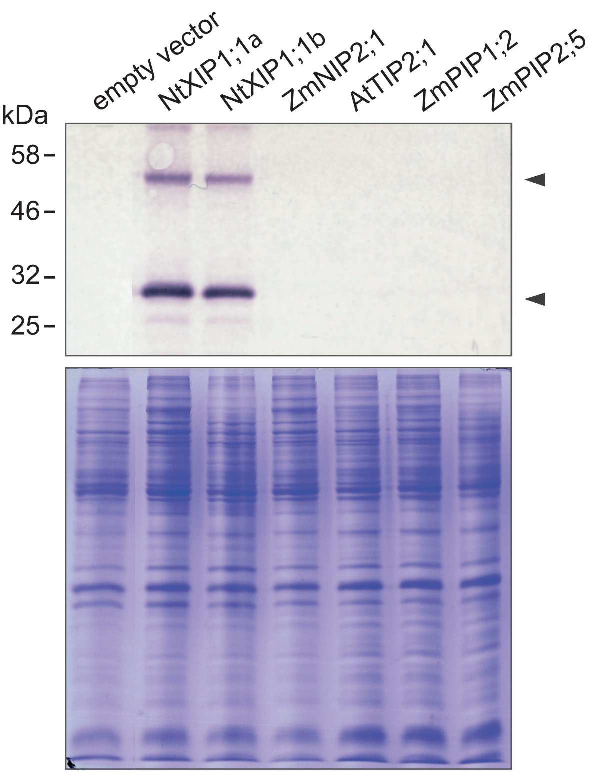


**Supplemental Figure S1. Characterization of the NtXIP1;1 antibody specificity**
Western blotting on microsomal membrane fractions of *S. cerevisiae*, expressing indicated isoforms of different aquaporin subfamilies of *Nicotiana tabacum* (Nt), *Zea mays* (Zm) or Arabidopsis (At) or an empty vector control using the generated polyclonal NtXIP1;1 antibody (upper picture) and a coomassie staining of the corresponding protein extracts (lower picture). Black arrowheads indicate the monomeric and dimeric versions of NtXIP1;1.

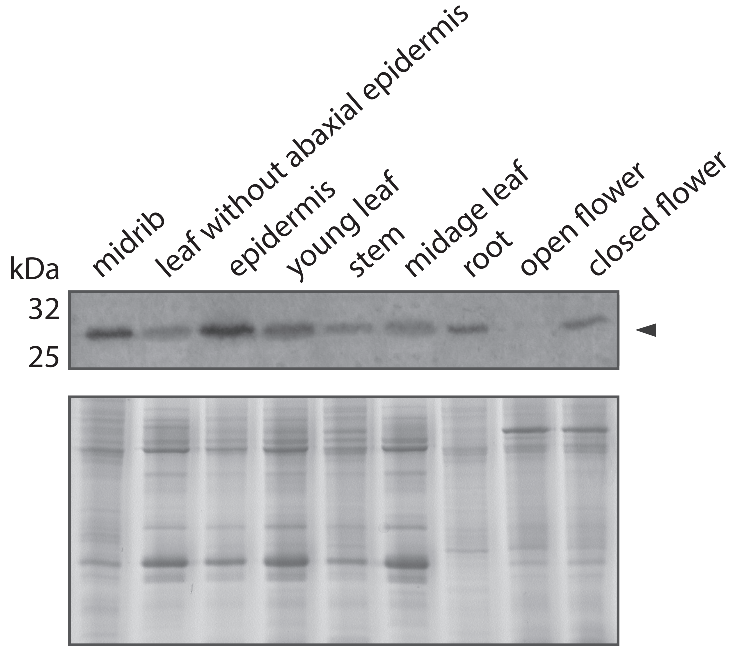


**Supplemental Figure S2. NtXIP1;1 protein expression in various *N. tabacum* tissues.** Western blotting on microsomal membrane fractions of various *N. tabacum* tissues using the NtXIP1;1 antibody (Supplemental Figure S1) (upper picture) and a coomassie staining of the corresponding protein extracts (lower picture). Above ground organs were sampled of five week old N. tabacum plants whereas the root material was taken from growth on synthetic medium of 2 week old plantlets. Black arrowhead indicates the monomeric NtXIP1;1.

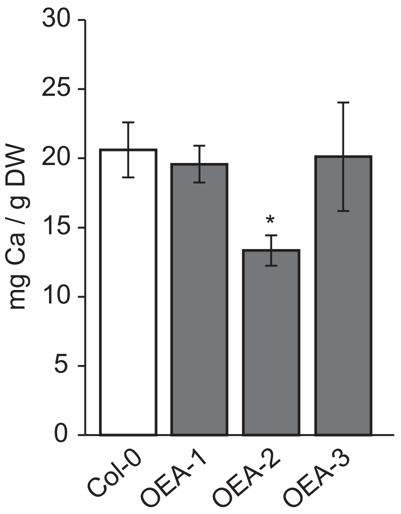


**Supplemental Figure S3. Calcium tissue concentrations of Arabidopsis plants overexpressing NtXIP1;1**

Calcium concentrations in leaves of wild type (Col-0) and NtXIP1;1 overexpressing lines (OE1-3). Chart bars represent means ± SD (n=4). Significant differences in leaf Ca concentrations compared to Col-0 were assessed (*$p<0.05$; Students *t*-test).

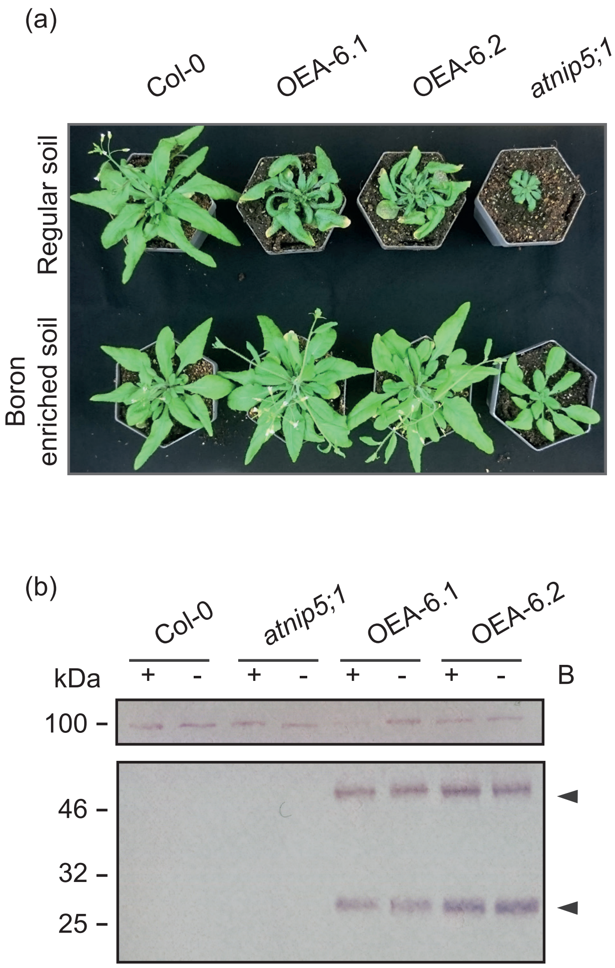


**Supplemental Figure S4. Boron deficiency of NtXIP1;1 overexpressing Arabidopsis lines can be rescued by boron fertilization**

(A) Wild type Arabidopsis (Col-0) and NtXIP1;1 overexpression line OEA-6 were grown on standard Arabidopsis soil substrate or the same soil substrate supplemented up to 2.4 mg B / kg soil (1 Liter of 200 µM $B(OH)_3$ per kg soil). Rosettes of plants with the same age are compared.

(B) Western blotting of leaf microsomal membrane fractions of wild type (Col-0) and NtXIP1;1 overexpression line OEA-6 grown under standard (-B) and B surplus (+B) soil substrate using the anti-NtXIP1;1 antibody. A general $H^+$-ATPase antibody (Morsomme et al., 1998) was used as a protein loading reference (upper blot). Black arrowheads indicate the monomeric and dimeric versions of NtXIP1;1 (lower blot).

**Morsomme, P., Dambly, S., Maudoux, O., and Boutry, M.** (1998). Single point mutations distributed in 10 soluble and membrane regions of the *Nicotiana plumbaginifolia* plasma membrane PMA2 $H^+$-ATPase activate the enzyme and modify the structure of the C-terminal region. J. Biol. Chem. **273**:34837–34842.

**Supplemental Table S1. Primers which have been used in this study.**

| | | |
|---|---|---|
| *NtXIP1;1* expression in *N. tabacum* | NtXIP1;1_fw | 5’ ATGGTACCATGGCTTCCAATGCTAGTCATG 3’ |
| | NtXIP1;1_rv | 5’ ATGGATCCTCATTCATGCAACCCAAACGAAG 3’ |
| *NtXIP1;1* silencing in *N. tabacum* | amiRNA2 I | 5’ GATAATTCCGACAAGCGCGGCGGTCTCTCTTTTGTATTCC 3’ |
| | amiRNA2 II | 5’ GACCGCCGCGCTTGTCGGAATTATCAAAGAGAATCAATGA 3’ |
| | amiRNA2 III | 5’ GACCACCGCGCTTGTGGGAATTTTCACAGGTCGTGATATG 3’ |
| | amiRNA2 IV | 5’ GATATCACTATGGTGTCCAACATTCTCTCTTTTGTATTCC 3’ |
| *NtXIP1;1* expression in *A. thaliana* | NtXIP1;1_fw | 5´ GGCTTAAUATGGCTTCCAATGCTAGTCATG 3´ |
| | NtXIP1;1_rv | 5´ GGTTTAAUTCATTCATGCAACCCAAACGAAG 3´ |
| *AtNIP5;1* promoter | AtNIP5;1p_fw | 5´ CACCTCGCTTCTCTTGGTCGATCA 3´ |
| | AtNIP5;1p_rv | 5´ CTCGAGTCCAACGTTTTTTTTTTTGGTTTTTTTTTTGG 3´ |
| *NtXIP1;1* behind the *AtNIP5;1* promoter | NtXIP1;1_fw | 5' GAGAGACTCGAGTGGAAATGGCTTCCAATGCTA 3' |
| | NtXIP1;1_rv | 5' CTCTCTGGCGCGCCTCATTCATGCAACCCAAACG 3' |
| *AtEF1α* | AtEF1α_fw | 5´ CCTTGGTGTCAAGCAGATGA 3´ |
| | AtEF1α_rv | 5´ TGAAGACACCTCCTTGATGATTT 3´ |
| *AtNIP5;1 genomic* | AtNIP5;1g_fw | 5´CACCGATTTTCCCTCTCCTGAT 3´ |
| | AtNIP5;1g_rv | 5´ GCATGCAGCGTTACCGATTA 3´ |
| *AtNIP5;1 T-DNA insertion* | AtNIP5;1g_fw | 5´CACCGATTTTCCCTCTCCTGAT 3´ |
| | Lba1 | 5´ TGGTTCACGTAGTGGCCATCG 3´ |
