# Supplementary material for: Overexpression of X Intrinsic Protein 1;1 in Nicotiana tabacum and Arabidopsis reduces boron allocation to shoot sink tissues
Source: Plant Direct. 2019 Jun 5;3(6):e00143. doi: 10.1002/pld3.143 (PMC6549384; doi:10.1002/pld3.143)
Supplement: Supplementary file 2 [file PLD3-3-e00143-s002.pdf]

Dear Dr. Bienert:

Thank you for submitting to Plant Direct. In light of the reviews transferred and my own evaluation, some editorial revisions are needed before the paper can be accepted for publication in Plant Direct. Please view the comments below and use them as a guide while you work on your revision.

When uploading the revised version of this article, please be sure to include the following:

-A word document that contains your response to the comments. You should respond to each comment and note the changes made to the manuscript. If you do not agree with a comment and choose not to make a suggested revision, please explain why. Please try to provide as complete an answer as possible to each comment.

-A tracked changes document with each change highlighted

- A clean version of the latest version of the manuscript

To upload your revision, please click the link below.

https://plantdirect.msubmit.net/cgi-bin/main.plex?el=A7Lr6Gq1A6FSr3I2A9ftdhK2w3zNoaNxdsJtafxJF9wZ

In order to provide as timely a service as possible, we ask that your revision is resubmitted within three months after receipt of this request. If an extension is needed, please send a request, along with a brief explanation, to the editorial office at plantdirect@wiley.com .

Please note that, in addition to publishing reviewer comments, the author's responses to review comments will also be published alongside the final version of the paper. If you would not like the author's responses to be published, please contact the editorial office at plantdirect@wiley.com .

Thank you very much for giving us an opportunity to review your work. I look forward to receiving the next version.

Sincerely,

Supervising Editor

Hsou-min Li

Dear Prof. Hsou-min Li,

We thank you for your very constructive and detailed review, comments and suggestions. We performed the requested changes and followed the suggestions, as it is specified below. We hope that our manuscript can now be accepted for publication in Plant Direct.

Sincerely, on behalf of the authors

Patrick Bienert and François Chaumont

Editor comments:

1. Please change your title from "Enhanced expression of X Intrinsic Protein 1;1 in ......" to "Over-expression of X Intrinsic Protein 1;1 in ....". The word "enhanced" implies that the "original" expression has been increased, but the over-expression promoters you used have different cell-type specificity from that of XIP1.

AUTHOR RESPONSE:

We changed the title accordingly.

2. Line 33: "Here, we demonstrated that overexpression of NtXIP1;1 in Nicotiana tabacum and in Arabidopsis, which does not contain any XIP gene, resulted in boron (B)-deficiency Symptoms"

Please specify the over-expression promoter used. For example "Here, we demonstrated that overexpression of NtXIP1;1 in Nicotiana tabacum and in Arabidopsis by the En2pPMA4 or the 35S CaMV promoter resulted in symptoms of boron (B)-deficiency". This detail is important because it is very likely that the observed phenotypes are related to the tissue- and cell-type-specificity of the promoters used. Accordingly, it would be useful for readers to have this information in the abstract.

AUTHOR RESPONSE:

We changed the text into: "Here, we demonstrated that overexpression of NtXIP1;1 in Nicotiana tabacum by the En2pPMA4 or the 35S CaMV promoter and in Arabidopsis, which does not contain any XIP gene, by the 35S CaMV promoter, resulted in boron (B)-deficiency symptoms such as death of the shoot apical meristem, infertile flowers, and puckered leaves."

3. Since young and old leaves often have different phenotypes and boron contents, please indicate the ages of the leaves used for the Western blots in the inset of Figure 1a. Please also indicate if the leaves were taken from plants of the same age as those shown in Figure 1a and specify the ages of those plants. The same information should also be added to Figure 3a.

AUTHOR RESPONSE:

For Figure 1a we used young leaves for blotting of five week old soil grown plants. We indicated in the Figure legend:

"Inset: Western blot showing the NtXIP1;1 protein level in young leaves of five week old soil grown WT, OE-1 and OE-2 lines detected using an anti-NtXIP1;1 antibody (upper picture), and coomassie blue staining of the corresponding microsomal membrane fraction (lower picture)."

The second sentence of Point 3 presumably refers to either Figure 1b or Figure 2a. We indicated the plant age (which was always the same) now also in both Figure legends as follows:

Figure 1b:

"Typical leaf tissue malformations and shoot apical meristem phenotypes observed in five to six week old tobacco lines overexpressing NtXIP1;1."

For Figure 2a we used leaves of plants of the same experimental set up as in Figure 1a. We stated this now in the Figure legend:

“Boron (upper panel) and calcium (lower panel) concentrations in different tissues of five week old wild type (WT) and NtXIP1;1 overexpressing OE-1 and OE-2 N. tabacum plant lines determined by ICP-MS analysis.”

For Figure 3a we used whole shoot samples of two week old plantlets. We indicated in the Figure legend:

(a) Western blotting of whole leaf microsomal fractions of two week old plantlets of N. tabacum wild type (WT) and amiRNA NtXIP1;1-silenced lines II-1.2 and II-2.2 using the NtXIP1;1-antibody demonstrating efficient silencing of NtXIP1;1 protein in the amiRNA lines (upper panel).

4. Line 430: "As for the B concentrations no significant changes in Ca concentrations were quantified (Figure 4a,b)."
Please change to "Similar to B concentrations, no significant changes in Ca concentrations were found (Figure 4a,b)."

AUTHOR RESPONSE:

We changed the text accordingly.

5. Line 437: "NtXIP1;1 was expressed in the root (data not shown), leaves, stem, and flowers (Figure 5a)."
It is journal policy not to allow statements supported by "data not shown". Please either provide the data on root expression or remove this statement.

AUTHOR RESPONSE:

We included a western blot showing protein expression in roots in the Supplemental Material (Supplemental Figure S2).

6. Line 444: "NtXIP1;1 protein levels correlated with the observed promoter activity and were higher in epidermal peels than in leaves lacking the abaxial epidermis."
Is this the same experiment as described in Figure 5b? If yes, please clearly indicate so or consider consolidating with the next paragraph describing Figure 5b.

AUTHOR RESPONSE:

The promoter activity refers to the results obtained in Bienert et al., 2011. We changed the text as follows to make our statement more precise:

NtXIP1;1 protein levels correlated with previously observed NtXIP1;1 promoter activity levels (Bienert et al., 2011) and were higher in epidermal peels than in leaves lacking the abaxial epidermis.

7. Line 548: "....overexpression of NtXIP1;1 in its endogenous but also in a heterologous plant background leads to B deficiency"
Add "in young leaves" to the end of this sentence.

AUTHOR RESPONSE:

We changed the text accordingly.

8. Line 591.......Remarkably, NtXIP1;1 translation was enriched in the leaf epidermis compared to... Change "translation" to "protein level".

AUTHOR RESPONSE:

We changed the text accordingly.

9. Please remove Figure 9 and its description.
From your response to previous reviewers, you seem to speculate that over-expression of XIP1 in roots results in "destroy(ing) boron gradient between the epidermis and the vascular system" and this is the reason for lower root-to-shoot boron transport in the over-expressing plants. This hypothesis is not shown in Figure 9, which was meant to explain the observed phenotypes. Moreover, this hypothesis is entirely speculative. In addition, Figure 9 suggests that the xylem of the shoot base in the over-expressing plants still has the same amount of boron, and "XIP1;1-overexpressors have high amounts of XIP1;1 proteins in all the plant tissues, disturbing the natural boron (B) fluxes and resulting in a progressive dilution of B in the vascular system". This is not supported by your data showing lower boron in xylem sap but the same amount of boron in older leaves in the over-expressing plants. Furthermore, the amounts of XIP1 present in different tissues (e.g. SAM vs. shoot base) in the over-expressing and knockdown lines were not investigated, but they have been drawn into Figure 9.

AUTHOR RESPONSE:

We removed Figure 9 from the manuscript.
